# Association between FABP2 Ala54Thr polymorphisms and type 2 diabetes mellitus risk: a HuGE Review and Meta-Analysis

**DOI:** 10.1111/jcmm.12385

**Published:** 2014-11-11

**Authors:** Chun-Jian Qiu, Xiao-Zheng Ye, Xiao-Juan Yu, Xiao-Ren Peng, Tong-Huan Li

**Affiliations:** a Department of Endocrinology, No. 81 Hospital of PLANanjing, China; b Department of Endocrinology, Jinling Hospital/Nanjing General Hospital of Nanjing Military Command, Nanjing University School of MedicineNanjing, China

**Keywords:** fatty acid-binding protein 2, single nucleotide polymorphism, diabetes mellitus, type 2, susceptibility, meta-analysis

## Abstract

Many studies have examined the association between the *FABP2* (rs1799883) Ala54Thr gene polymorphism and type 2 diabetes mellitus risk (T2DM) in various populations, but their results have been inconsistent. To assess this relationship more precisely, A HuGE review and meta-analysis were performed. The PubMed and CNKI database was searched for case-control studies published up to April 2014. Data were extracted and pooled odds ratios (OR) with 95% confidence intervals (CI) were calculated. Ultimately, 13 studies, comprising 2020 T2DM cases and 2910 controls were included. Overall, for the Thr carriers (Ala/Thr and Thr/Thr) *versus* the wild-type homozygotes (Ala/Ala), the pooled OR was 1.18 (95% CI = 1.04–1.34, *P* = 0.062 for heterogeneity), for Thr/Thr *versus* Ala/Ala the pooled OR was 1.17 (95% CI = 1.05–1.41 *P* = 0.087 for heterogeneity). In the stratified analysis by ethnicity, the significantly risks were found among Asians but not Caucasians. This meta-analysis suggests that the *FABP2* (rs1799883) Ala54Thr polymorphisms are associated with increased susceptibility to T2DM risk among Asians but not Caucasians.

## Introduction

Type 2 diabetes mellitus (T2DM) has elevated prevalence, morbidity and mortality rates and the social and economic repercussions of its chronic complications compromise both the quality of life and productivity of those affected, making it a serious public health problem [1,2]. Meanwhile, T2DM is a major factor that along with other risk factors such as obesity, endothelial dysfunction, and dyslipidemia contribute to atherosclerotic diseases. Although it results from synthetic action involving insulin resistance and impaired insulin secretion [3], a detailed aetiology underlying T2DM is still unclear.

Currently, more and more studies confirmed that T2DM was determined by the combined effects of multiple genetic and environmental factors [4]. The most likely explanation for the dramatic increase in T2DM prevalence observed is changing patterns of diet and physical activity (PA). However, it is believed that these environmental changes may only lead to T2DM in the presence of a permissive genetic background [4].

Fatty acid-binding protein 2 (*FABP2*) genes play a key role in the absorption and intracellular transport of dietary long chain fatty acids. The *FABP2* gene is located in the 4q28–4q31 chromosomal region, consists of ∼3.4 kbs [5] and codes for the intestinal fatty acid-binding protein (I-FABP). *FABP2* is a member of the FABPs superfamily that produces intracellular proteins which bind hydrophobic ligands reversibly [6,7]. The G to A polymorphism (rs1799883) of codon 54 results in the substitution of threonine (Thr) for alanine (Ala) [8,9].

Recently, many studies have investigated the role of the *FABP2* (rs1799883) Ala54Thr polymorphisms in T2DM. However, the results of these studies remain inconclusive. A single study might not be powered sufficiently to detect a small effect of the polymorphisms on T2DM, particularly in relatively small sample sizes. Various types of study populations might also have contributed to these disparate findings. To clarify the effect of the *FABP2* (rs1799883) Ala54Thr polymorphism on the risk for T2DM, we performed a meta-analysis of all eligible case-control studies that have been published.

## Materials and methods

### Publication search

We searched for studies in the PubMed and CNKI (China National Knowledge Infrastructure) electronic databases to include in this meta-analysis, using the terms ‘fatty acid-binding protein 2’, ‘*FABP2*’ ‘polymorphism’ ‘type 2’ and ‘diabetes mellitus’. An upper date limit of 1 April 2014 was applied; no lower date limit was used. The search was performed without any restrictions on language and was focused on studies that had been conducted in humans. We also reviewed the Cochrane Library for relevant articles. Concurrently, the reference lists of reviews and retrieved articles were searched manually. Only full-text articles were included. When the same patient population appeared in several publications, only the most recent or complete study was included in this meta-analysis.

### Inclusion criteria

For inclusion, the studies must have met the following criteria: they (*i*) evaluated *FABP2* Ala54Thr polymorphisms and T2DM risk; (*ii*) were case-control studies; (*iii*) supplied the number of individual genotypes for the *FABP2* Ala54Thr polymorphisms in T2DM cases and controls respectively; and (*iv*) demonstrated that the distribution of genotypes among controls were in Hardy–Weinberg equilibrium.

### Data extraction

Information was extracted carefully from all eligible publications independently by two authors, based on the inclusion criteria above. Disagreements were resolved through a discussion between the two authors.

The following data were collected from each study: first author's surname, year of publication, ethnicity, total numbers of cases and controls, and numbers of cases and controls who harboured the *FABP2* Ala54Thr genotypes respectively. If data from any category were not reported in the primary study, the items were designated ‘not applicable’. We did not contact the author of the primary study to request the information. Different ethnicity descents were categorized as Asian and Caucasian. We did not require a minimum number of patients for a study to be included in our meta-analysis.

### Statistical analysis

Odds ratios (ORs) with 95% CIs were used to determine the strength of association between the *FABP2* Ala54Thr polymorphisms and T2DM risk. The pooled ORs for the risk associated with the *FABP2* Ala54Thr genotype, the Thr carriers (Ala/Thr and Thr/Thr) *versus* the wild-type homozygotes (Ala/Ala), Thr/Thr *versus* Ala/Ala were calculated respectively. Subgroup analyses were done by ethnicity. Heterogeneity assumptions were assessed by chi-square-based *Q*-test [10]. A *P-*value greater than 0.10 for the *Q*-test indicated a lack of heterogeneity among the studies. Thus, the pooled OR estimate of each study was calculated by using the fixed-effects model (the Mantel–Haenszel method) [11]; otherwise, the random-effects model (the DerSimonian and Laird method) was used [12].

One-way sensitivity analyses were performed to determine the stability of the results-each individual study in the meta-analysis was omitted to reflect the influence of the individual dataset on the pooled OR [13].

Potential publication biases were estimated by funnel plot, in which the standard error of log (OR) of each study was plotted against its log (OR). An asymmetrical plot suggests a publication bias. Funnel plot asymmetry was assessed by Egger's linear regression test, a linear regression approach that measures the funnel plot asymmetry on a natural logarithm scale of the OR. The significance of the intercept was determined by *t*-test, as suggested by Egger (*P* < 0.05 was considered a statistically significant publication bias) [14].

All calculations were performed with STATA, version 10.0 (Stata Corporation, College Station, TX, USA).

## Results

### Study characteristics

A total of thirteen publications involving 2020 T2DM cases and 2910 controls met the inclusion criteria and were ultimately analysed [15–27]. Table 1 presents the main characteristics of these studies. Among the 13 publications, 11 were published in English and the others were published in Chinese. The sample sizes ranged from 100 to 923. There were 6 groups of Asians and 7 groups of Caucasians. Seven studies were hospital-based case-control studies and six were population-based case-control studies. All polymorphisms in the control participants were in Hardy–Weinberg equilibrium.

**Table 1:** Distribution of FABP2 Ala54Thr genotypes among T2DM cases and controls included in this meta-analysis

First author-year	Ethnicity (country of origin)	Total sample (case/control)	T2DM cases			Controls		
	
			Ala/Ala	Ala/Thr	Thr/Thr	Ala/Ala	Ala/Thr	Thr/Thr
Alharbi-2014	Asian (Saudi Arabia)	438/460	220	170	37	260	171	29
Raza-2013	Asian (India)	190/110	35	127	28	25	68	17
Shi-2012	Asian (China)	117/108	32	64	21	46	48	14
Bu-2011	Caucasian (USA)	202/130	116	86*		79	51*	
Tavridou-2009	Caucasian (Greece)	242/188	104	104	24	104	71	13
Albala-2007	Caucasian (Chilean)	70/216	14	56*		61	155*	
Li-2006	Caucasian (Germany)	192/384	66	87	35	114	182	80
Guettier-2005	Asian (India)	105/65	51	54*		29	36*	
Duarte-2003	Caucasian (Australia)	104/819	82	22*		625	194*	
Wang-2001	Asian (China)	102/102	53	38	11	49	44	9
Ito-1999	Asian (Japan)	150/147	51	76	23	63	62	22
Boullu-Sanchis-1999	Caucasian (France)	89/100	34	33	22	53	33	14
Rissanen-1997	Caucasian (Finnish)	19/81	13	6*		39	42*	

* The number of the combined Ala/Thr and Thr/Thr genotypes.

Ala/Ala indicates wild-type, Ala/Thr indicates heterozygote, Thr/Thr indicates variant homozygote.

### Meta-analysis results

**Fig. 1:**
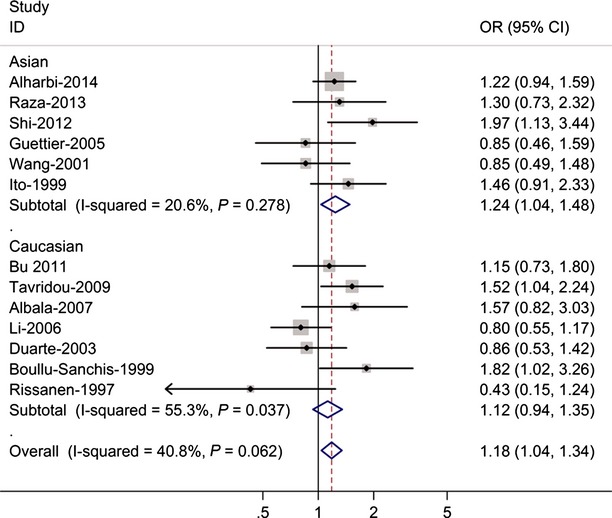
Forest plot (random-effects model) of T2DM risk associated with FABP2 Ala54Thr polymorphisms and T2DM risk for the (Ala/Thr and Thr/Thr) *versus* Ala/Ala. Each box represents the OR point estimate, and its area is proportional to the weight of the study. The diamond represents the overall summary estimate, with CI represented by its width. The vertical line is set at the null value (OR = 1.0).

Table 2 listed the main results of this meta-analysis. Overall, the Thr carriers (Ala/Thr and Thr/Thr) *versus* the wild-type homozygotes (Ala/Ala), the pooled OR for all studies combined 2020 T2DM cases and 2910 controls was 1.18 (95% CI = 1.04–1.34, *P* = 0.062 for heterogeneity; Fig. 1), for Thr/Thr *versus* Ala/Ala the pooled OR was 1.17 (95% CI = 1.05–1.41 *P* = 0.087 for heterogeneity).

**Table 2:** Summary ORs for various contrasts of FABP2 Ala54Thr polymorphisms in this meta-analysis

	Number of studies	OR (95% CI) *P* (*Q*-test)	
	
		(Ala/Thr and Thr/Thr) *versus* Ala/Ala	Thr/Thr *versus* Ala/Ala
Total	13	1.18 (1.04–1.34) 0.062	1.17 (1.05–1.41) 0.087
Caucasian	7	1.12 (0.94–1.35) 0.037	1.09 (0.89–1.39) 0.033
Asian	6	1.24 (1.04–1.48) 0.278	1.29 (1.08–1.49) 0.256

*P* (*Q*-test), *P*-value of *Q*-test for heterogeneity test; OR, odds ratio; CI, confidence interval.

In the stratified analysis by ethnicity, significantly risks were found among Asians for (Ala/Thr and Thr/Thr) *versus* (Ala/Ala) (OR = 1.24, 95% CI = 1.04–1.48; *P* = 0.278 for heterogeneity) and Thr/Thr *versus* Ala/Ala (OR = 1.29; 95% CI = 1.08–1.49; *P* = 0.256 for heterogeneity). However, for Caucasians, significantly risks were not found for (Ala/Thr and Thr/Thr) *versus* (Ala/Ala; OR = 1.12, 95% CI = 0.94–1.35; *P* = 0.037 for heterogeneity) and Thr/Thr *versus* Ala/Ala (OR = 1.09; 95% CI = 0.89–1.39; *P* = 0.033 for heterogeneity).

### Sensitivity analyses

A single study involved in the meta-analysis was deleted each time to reflect the influence of the individual data set to the pooled ORs, and the corresponding pooled Ors were not materially altered (data not shown).

### Publication bias

**Fig. 2:**
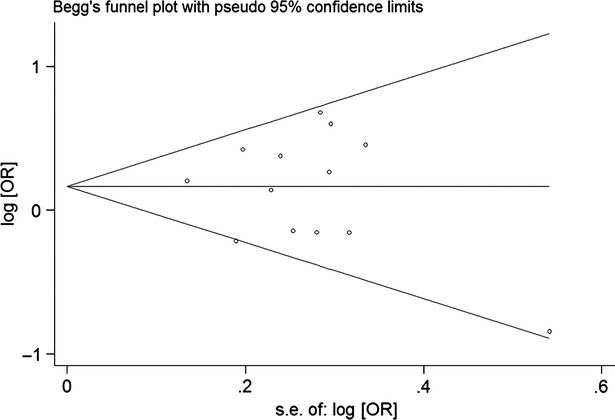
Begg's funnel plot of FABP2 Ala54Thr polymorphisms and T2DM risk for the (Ala/Thr and Thr/Thr) *versus* Ala/Ala.

Begg's funnel plot and Egger's test were performed to access the publication bias of literatures. Evaluation of publication bias for (Ala/Thr and Thr/Thr) *versus* (Ala/Ala) showed that the Egger test was not significant (*P* = 0.700; Fig. 2). Meanwhile, for Thr/Thr *versus* Ala/Ala the publication bias was not found (*P* = 0.214). These results did not indicate a potential for publication bias.

## Discussion

The *FABP2* gene has been proposed as a candidate gene for diabetes and insulin resistance because the protein it encodes is involved in fatty acid absorption and metabolism [28]. Numerous studies have assessed *FABP2* gene variants and their association with insulin resistance and T2DM. The most studied variant is the Ala54Thr variation at codon 54, a mis-sense variant that has a definite effect on the primary structure of the protein and affects its fatty acid-binding properties. Previous studies have found a significant association between the *FABP2* genotype and occurrence of T2DM or decreased insulin sensitivity [8]. In Pima Indians, *FABP2* Ala54Thr genotypes was shown to be associated with increased fatty acid-binding, increased fat oxidation, and insulin resistance [8]. On the other hand, in Finnish non-diabetic and NIDDM participants [27], the Ala54Thr polymorphism of the *FABP2* gene did not influence insulin sensitivity.

To our knowledge, the current meta-analysis firstly elucidated the potential association between the *FABP2* Ala54Thr polymorphisms and T2DM risk based on a Human Genome Epidemiology review of all research published up to April, 2014. This meta-analysis explored the association between the *FABP2* Ala54Thr polymorphisms and T2DM risk. Our results indicated that *FABP2* Ala54Thr polymorphism was significantly associated with the susceptibility to T2DM among Asians but not in Caucasians.

Population stratification is a troubling issue and can lead to spurious evidence on the association between biomarkers and a disease, implicating the disparate effects of environment and ethnic differences on genetic background [29]. In this meta-analysis, ethnicity stratification of differences between Asians and Caucasians was found. Meanwhile, because the same polymorphism seemed to play different roles in T2DM susceptibility among different ethnic populations and because the frequencies of single nucleotide polymorphisms were different among different ethnic groups, subgroup analyses based on ethnicity were conducted. The Thr allele frequencies for FABP2 Ala54Thr polymorphism described in different populations between in Pima Indians (30%), Korean(34%), Japanese (35%), Swedish (30%) and Caucasian individuals from USA (32%).[8,30–33]. In the included studies of our meta-analysis, the Thr allele frequencies for FABP2 polymorphism fluctuated form 28–34%.

It is important to keep in mind that type 2 diabetes is clearly multifactorial in origin, being determined by both genetic and environmental factors. Body composition, habitual PA levels, and diet are factors that are at least partly environmentally determined, and all are known to exert their own substantial and independent effects on insulin resistance and type 2 diabetes. Environmental factors clearly must play a substantial role in determining type 2 diabetes prevalence. During this same time, factors such as habitual PA levels and body composition have changed substantially, while negligible genetic alterations could have occurred over this time frame. Multiplegene/environment interactions [28,34] would determine a potential effect between FABP2 Ala54Thr polymorphism and T2DM risk. Glycemia in diabetic mellitus patients are extremely variables depending on diet, PA and pharmacotherapy adherence.

Zhao *et al*. study [35] performed meta-analysis to explore the association between *FABP2* Ala54Thr polymorphism and insulin resistance and blood glucose. They found that *FABP2* Ala54Thr polymorphism was weakly associated with a higher degree of insulin resistance, higher level of fasting insulin and higher level of 2-h BG. However, they did not perform the meta-analysis on the association of the *FABP2* Ala54Thr polymorphism with risk of type 2 diabetes because of few studies on this association. In addition, that meta-analysis did not perform subgroup analyses by ethnicity. Since then, several additional studies with large cohort populations have been reported. We have performed this meta-analysis by including more recent related studies and by generally by using a more comprehensive search strategy. Study screening and data extraction were performed independently and reproducibly by two reviewers. Moreover, we also explored the association between the *FABP2* Ala54Thr polymorphisms and T2DM risk for subgroups divided according ethnicity stratification.

Some limitations of this meta-analysis should be acknowledged. First, heterogeneity can interfere with the interpretation of the results of a meta-analysis. Although we minimized this likelihood by performing a careful search of published studies, by using explicit criteria for a study's inclusion and performing strict data extraction and analysis, significant interstudy heterogeneity nevertheless existed in nearly every comparison. The presence of heterogeneity can result from differences in the selection of controls, age distribution, and prevalence of lifestyle factors. Although most controls were selected from healthy populations, some studies had selected controls among friends or family members of lung cancer patients or patients with other diseases. Further, only published studies were included in this meta-analysis. The presence of publication bias indicates that non-significant or negative findings might be unpublished. Finally, our results were based on unadjusted estimates; a more precise analysis should have been conducted if individual data were available, which would have allowed us to adjust using other covariates, including age, ethnicity, family history, environmental factors and lifestyle.

Despite these limitations, this meta-analysis suggests that the *FABP2* Ala54Thr polymorphisms are associated with the increased susceptibility to T2DM risk among Asians but not Caucasians. Large-sample studies of different ethnic groups with carefully matched cases and controls should be considered in future association studies to confirm the results of our meta-analysis.
